# A Rare Component of Psammomatous Meningioma in a Testicular Teratoma

**DOI:** 10.1155/2013/645415

**Published:** 2013-11-10

**Authors:** Fábio Meira Castro Pereira, Marbele Guimarães de Oliveira, Laiana do Carmo Almeida, Bruno Cunha Pires, José de Bessa Júnior

**Affiliations:** ^1^Department of Anatomic Pathology, Universidade Estadual de Feira de Santana (UEFS), Medical School, BR-116, Km 3, Campo Limpo, 44054-008 Feira de Santana, BA, Brazil; ^2^Division of Urology, Department of Surgery, Universidade Estadual de Feira de Santana (UEFS), Medical School, BR-116, Km 3, Campo Limpo, 44054-008 Feira de Santana, BA, Brazil

## Abstract

We report a case of a psammomatous variant of meningioma arising in a pure and mature testicular teratoma. At immunohistochemistry, the meningiomatous component showed epithelial membrane antigen, S100 protein, and vimentin positive. Benign neoplasms arising in testicular teratomas are extremely rare. To our knowledge, we have not found any such report of psammomatous variant of meningioma in a testicular teratoma and any meningioma arising in a pure and mature testicular teratoma. This is a unique phenomenon.

## 1. Introduction

Teratomas are composed of several types of tissues representing different germinal layers (endoderm, mesoderm, and ectoderm). They may be composed exclusively of well-differentiated, mature tissues or have immature, fetal-like tissues [1]. Malignant transformation of teratomas, indicating the presence of a nongerm cell malignancy, has been well documented in testes; on the other hand, benign neoplasms appearing in teratoma are very rare. We report a curious case of psammomatous variant of meningioma arising in a pure and mature testicular teratoma.

## 2. Case Report

**Figure 1:**
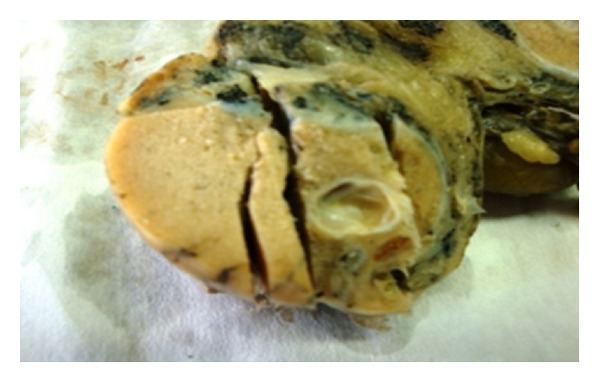
Macroscopic appearance of the surgical specimen. A solid-cystic mass with areas of calcification measuring 2.0 cm in testicular parenchyma.

*Case Presentation*. A 46-year-old man presented with right testicular enlargement. Ultrasonography performed at an outside institution showed solid-cystic testicular mass with calcifications. A right radical orchiectomy and follow-up were peformed at the outside institution. On gross description, the right orchiectomy specimen consisted of a testis measuring 5.5 × 2.5 × 3.0 cm in dimension. A solid-cystic mass with areas of calcifications measuring 2.0 cm was identified within the testicular parenchyma (Figure 1). The mass was limited to testis and it did not appear to invade tunica albuginea, tunica vaginalis, spermatic cord, and epididymis. It was sampling 1 cm^2^ section for every centimetre of maximum tumor diameter.

**Figure 2:**
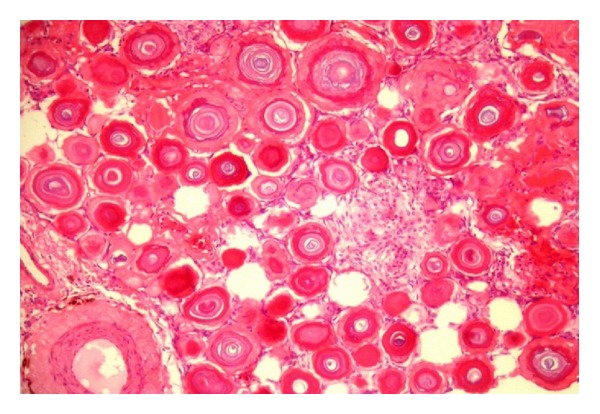
Microscopic appearance of the surgical specimen. Numerous psammomatous bodies admixed among fusiform meningothelial cells (H&E).

**Figure 3:**
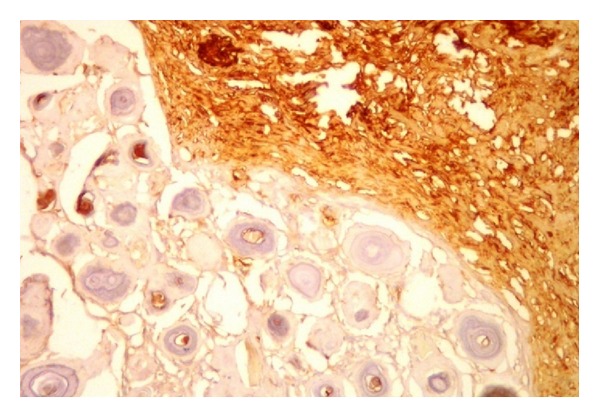
Positive immunohistochemical staining of meningothelial cells, epithelial membrane antigen.

**Figure 4:**
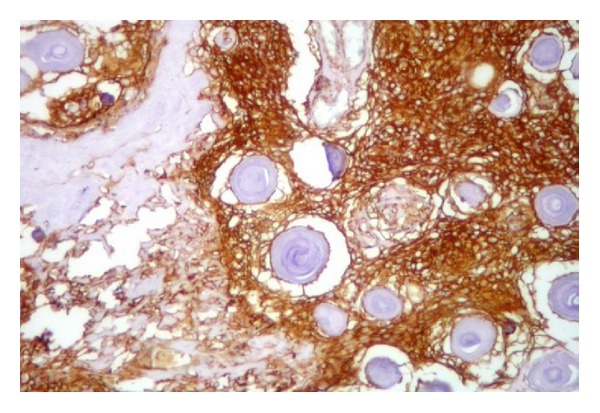
Immunohistochemical staining positive for vimentin in meningothelial cells.

Histologic sections of the surgical specimen showed a teratoma composed of mature tissues epithelial-lined cystic spaces, solid areas of nervous tissue, blood vessels, and a component of numerous psammomatous bodies confluent admixed among fusiform meningothelial cells. These cells had a morphologic appearance consistent with the psammomatous variant of meningioma (Figure 2). Therefore, this component was identified as a benign neoplasm arising in teratoma. Immunohistochemical staining supported this diagnosis with strong and diffusely positive staining for epithelial membrane antigen and vimentin and focal areas of positive staining for S100 protein (Figures 3 and 4). The meningiomatous component was found entirely within the confines of the tumor mass and did not spread to the testicular or extratesticular tissue. There was no evidence of microscopic vascular or lymphatic invasion by tumor cells.

## 3. Discussion

From the tissues that comprise teratomas may arise benign and malignant neoplasms. Malignant transformation of teratoma components can be seen in 3–6% of metastatic germ cell tumors [2] and has been well documented, most commonly, in ovary [3, 4].

It is important to recognize these malignant tumors that arise in teratomas because of the prognostic importance of their identification. Patients in whom the malignant component is localized to the testis do well, but patients in whom the nongerm cell component metastasizes do poorly [5].

Reports of secondary benign tumors arising in teratomas are uncommon and consist of a scattering of case reports. To date, accounts of blue nevi, prolactinoma, epithelioid hemangioma, and sebaceous adenoma have been described as arising in teratomas of the ovary [6].

Secondary benign neoplasm in testicular teratomas is extremely rare. There is a single case report in the literature of a microcystic variant of meningioma developed in mixed germ cell testicular teratoma composed predominantly of mature and immature tissues, with elements of seminoma and carcinoma embrionario [6].

To our knowledge, this is the second case report of meningioma arising in a testicular teratoma and the first of psammomatous variant occurring in a pure and mature teratoma. This case, therefore, adds to the medical literature an interesting and distinct case of a benign tumor originating in a teratoma testicular and reaffirm, teratomas develop from totipotential germ cells, and consequently contain all three germ cell layers as well as neoplasm of all of these tissues.

It is important to recognize neoplasms arising in teratomas, because some of them may have adverse clinical behavior, especially malignant components not restricted to the testis. In this case, we observe a rare benign component that represents a curiosity.
